# A Sensitive Near-Infrared Fluorescent Probe for Detecting Heavy Metal Ag^+^ in Water Samples

**DOI:** 10.3390/s19020247

**Published:** 2019-01-10

**Authors:** Yawen Zhang, Aiying Ye, Yuewei Yao, Cheng Yao

**Affiliations:** 1 College of Chemistry and Molecular Engineering, Nanjing Tech University, Nanjing 210000, China; 18761605638@163.com (Y.Z.); 8000000683@czie.edu.cn (A.Y.); yaoyw89@163.com (Y.Y.); 2 Changzhou Vocational Institute of Engineering, Changzhou 213100, China

**Keywords:** fluorescence probe, Ag^+^, sensitivity, selectivity, water samples

## Abstract

Silver is a common catalyst in industrial production, and the frequent use of Ag^+^ can cause water pollution. Thus, the detection of Ag^+^ in the environment is necessary to determine the level of pollution from silver. In this work, we designed a new, highly selective near-infrared (NIR) fluorescent probe QCy to detect Ag^+^. The probe exhibits “turn-off” fluorescence quenching responses at 760 nm towards Ag^+^ over other relevant cations, with outstanding sensitivity and a low detection limit (0.03 µM), which is considerably lower than the standard of the World Health Organization (WHO) for drinking water (0.9 µM). Meanwhile, QCy showed a very good linearity at a low concentration of Ag^+^ with a ‘naked eye’ visible color change of solution from blue to red. The probe has been applied successfully for the detection of Ag^+^ in real water samples.

## 1. Introduction

Silver is a widely used resource in various fields, such as electronics manufacturing, pharmaceuticals, photography, and imaging [1]. Unfortunately, such use can release large amounts of Ag^+^ into the environment, leading to the pollution of water sources, and threats to food and agricultural safety [2]. Several studies have reported the harmful effects of Ag^+^ on human health and the environment [3,4]. Ag^+^ is considered to be a very dangerous heavy metal pollutant [5,6]. Hence, designing a method to detect Ag^+^ with high selectivity, sensitivity, and efficiency is necessary [7,8,9].

Many traditional instrumental methods can be used to detect Ag^+^, including coupled plasma mass spectrometry, atomic absorption spectrometry, and stripping voltammetry [10,11]. However, although these methods show good accuracy and resolution, they are rather expensive and complicated procedures [12,13]. Fluorescence detection methods have attracted increasing attention in the past few years, because they present the advantages of high selectivity and sensitivity, and they can be applied to real-time detection. As such, fluorescence detection methods are more convenient than traditional instrumental methods [14,15,16,17]. Several fluorescent probes for detecting Ag^+^ have been developed. For example, Zhang et al. reported that compounds with two tetraphenylethylenes bearing adenine and thymine moieties were fluorescence turn-on chemosensors for Ag^+^ and Hg^2+^ [18] and Lee et al. reported an NS_2_O_2_-macrocycle-based fluoroionophore as a highly selective turn-on-type fluorescence chemosensor for Ag^+^ in a 1:1 (*v*/*v*) aqueous ethanol solution with a detection limit of 0.22 µM [19]. However, these probes are not sensitive enough to detect Ag^+^ in water samples. In addition, it is difficult for some of the reported probes in the literatures to discriminate Ag^+^ from other metal ions such as Hg^2+^ (Table S1) [20,21]. Therefore, it is significant to develop new fluorescent probes that can detect Ag^+^ sensitively and selectively in real samples.

The heptamethine cyanine-based dye possesses excellent spectral properties, such as high molar absorptivity and fluorescence quantum yield and good stability. Thus, it is an ideal fluorophore for a fluorescent probe. To date, cyanine-based near-infrared fluorescent probes are mainly used in biological analyses for nucleic acid staining or labeling, amino acid, and peptide and protein derivation or labeling. There are few reports about cyanine-based fluorescent probes for detecting silver ions. Based on the intramolecular d–π interactions, we designed and synthesized the heptamethine cyanine dye-based probe QCy to detect silver ions. The purpose of the introduction of an amine group is to increase the chelation of the probe and the silver ion. The probe shows fluorescent quenching (turn-off) of about 60-fold when 7 µM Ag^+^ is added in to an ethanol–phosphate-buffered saline (EtOH/PBS) (1:9) mixture. The detection limit (0.03 µM) is low, which matches the allowable level of Ag^+^ in drinking water by WHO [22]. QCy showed high selectivity to Ag^+^ over other metal cations. In addition, this probe can detect trace quantities of Ag^+^ in water samples.

## 2. Experimental Section

### 2.1. Materials

All of the solvents used in this work were of analytical purity, and the materials required were obtained from commercial suppliers or prepared by our laboratory without further purification of the commercially supplied chemicals before use. Deionized water was used for all measurements. All spectrographic measurements were performed in 10 mM phosphate buffered saline (PBS) (pH = 7.0). Cationic salts (FeCl_2_·4H_2_O, KNO_3_, NaNO_3_, Ca(NO_3_)_2_·4H_2_O, Zn(NO_3_)_2_·6H_2_O, Mg(NO_3_)_2_·6H_2_O, Pb(NO_3_)_2_, Ni(NO_3_)_2_·6H_2_O, Hg(NO_3_)_2_·8H_2_O, MnCl_2_·4H_2_O, CdNO_3_·4H_2_O, Cu(NO_3_)_2_·3H_2_O, and AgNO_3_) were purchased from Shanghai Ling Feng Chemical Co., Ltd. (Shanghai, China). All other chemical reagents used were of analytical grade and purchased from Sinopharm Chemical Reagent Co., Ltd. (Shanghai, China).

### 2.2. Instruments

A Sartorius PB-10 basic pH meter (INESA, Shanghai, China) was used for pH determination. ^1^H NMR and ^13^C NMR spectra were measured using a BrukerAV-400 spectrometer (Bruker, Karlsruhe, Germany) with chemical shifts reported in ppm, (in CDCl_3_ or Dimethyl sulfoxide-d_6_ (DMSO-d_6_); Tetramethylsilane (TMS) as an internal standard). Electrospray ionization mass spectra (ESI-Mass) were measured on a Micromass LCTTM system (Thermo Fisher, Shanghai, China), UV-vis spectra were recorded on a Shimadzu UV-2501 spectrometer (Shimadzu, Beijing, China), and fluorescence spectra were measured on a Perkin Elmer LS50B fluorescence spectrometer (PerkinElmer, Shanghai, China) at room temperature.

### 2.3. Synthesis

#### 2.3.1. Synthesis of 1-Ethyl-2,3,3-trimethyl-3H-indol-1-ium Iodide (**1**)

Compound **1** was synthesized by using the method described in the literature, with slight improvements [23]. Briefly, 2,3,3-trimethylindolenine (32 mmol; 5.1 g) and idoethane (96 mmol; 14.8 g) were dissolved in CH_3_CN (75 mL). The mixture was refluxed for 24 h, and then removed from heat. The solvent was removed under vacuum, and the crude product was dissolved in a small amount of acetone. Finally, the mixture was precipitated with cold ether, filtered, and dried in vacuum to obtain a pink product (9.57 g, yield: 95%). ^1^H NMR (400 MHz, chloroform-d) δ: 7.71 (d, J = 4.7 Hz, 1H), 7.55 (q, J = 5.5, 4.6 Hz, 3H), 4.69 (q, J = 7.4 Hz, 2H), 3.10 (s, 3H), 1.60 (d, J = 8.8 Hz, 9H). ^13^C NMR (100 MHz, chloroform-d) δ: 195.33, 141.61, 140.45, 130.15, 129.53, 123.50, 115.36, 54.65, 45.33, 23.09, 16.97, 13.72. ESI-MS: *m*/*z* calculated for C_13_H_18_N^+^: 188.14; found: 188.1.

#### 2.3.2. Synthesis of 2-Chloro-3-(hydroxymethylene)-1-cyclohexene-carboxaldehyde (**2**)

Compound **2** was prepared according to a previously reported method [24]. A solution of phosphorus oxychloride (4.3 mL) in dichloromethane (DCM) (3 mL) was added dropwise with stirring to an anhydrous solution of dimethylformamide (DMF) (5 mL) in an ice bath. The reaction mixture was continuously stirred for 30 min. Then, 1.04 g of cyclohexanone in 1 mL of DCM was added dropwise into the mixture; the solution color immediately changed from colorless to yellow. This mixture was refluxed for 3 h, poured into 200 mL of ice water, and allowed to stand overnight in a refrigerator. Thereafter, the mixture was filtered and dried in vacuum to obtain a yellow solid (0.97 g, yield: 93%). No further purification was performed, and the solid kept in a refrigerator for the next reaction steps.

#### 2.3.3. Synthesis of Compound Cy7-Cl

Cy7-Cl was synthesized using a method similar to that described in a previous report, with slight improvements [25]. Compounds **1** (1.6 g, 5 mmol), **2** (0.348 g, 2 mmol), and anhydrous sodium acetate (0.41 g, 5 mmol) were dissolved in 15 mL of acetic anhydride in a 50 mL round-bottomed flask, under a N_2_ atmosphere. The solution was heated to 100 °C for 1 h and stirred for 1 h, during which the solution color changed from red to dark green. The mixture was cooled to room temperature and then poured into 200 mL of diethyl ether to remove the acetic anhydride by filtration. The solid was washed with saturated brine (150 mL) and extracted with methylene chloride (3 × 200 mL). The extract was dried over anhydrous Na_2_SO_4_ to evaporate the solvent, yield a dark green solid (Cy7-Cl), 0.87 g, yield: 84%. ^1^H NMR (400 MHz, chloroform-d) δ: 8.36 (d, J = 14.1 Hz, 2H), 7.00 (d, J = 7.5 Hz, 4H), 7.30–7.25 (m, 2H), 7.20 (d, J = 7.8 Hz, 2H), 6.21 (d, J = 14.1 Hz, 2H), 4.26 (q, J = 7.2 Hz, 4H), 2.75 (t, J = 6.1 Hz, 4H), 2.03–1.95 (m, 2H), 1.72 (s, 12H), 1.47 (t, J = 7.2 Hz, 6H). ^13^C NMR (100 MHz, chloroform-d) δ: 171.89, 150.64, 144.50, 141.67, 141.09, 128.87, 127.19, 125.37, 122.34, 110.80, 100.84, 49.36, 40.07, 28.06, 26.69, 20.67, 12.49. ESI-MS: *m*/*z* calculated for C_34_H_40_ClN_2_^+^: 511.29; found: 511.3.

#### 2.3.4. Synthesis of Compound QCy

Cy7-Cl (119 mg, 0.25 mmol) was dissolved in 50 mL of anhydrous acetonitrile in a 50 mL round-bottomed flask, followed by addition of ethylamine in tetrahydrofuran (THF) solution (2 M, 1.5 mL). The solution was protected from light and heated to 50 °C, during which its color changed from green to blue. After removal of the solvent, the residue was dissolved in DCM, and purified by silica gel chromatography with methanol/DCM (1:99 to 5:95) as the eluent, to afford the product QCy as a blue solid (0.11 g, yield: 87%). ^1^H NMR (400 MHz, chloroform-d) δ: 7.70 (d, J = 10.0 Hz, 2H), 7.25 (d, J = 7.2 Hz, 4H), 7.04 (t, J = 6.9 Hz, 2H), 6.85 (d, J = 7.2 Hz, 2H), 5.58 (d, J = 11.3 Hz, 2H), 3.88 (d, J = 16.5 Hz, 6H), 2.48 (s, 4H), 1.86–1.78 (m, 3H), 1.68 (s, 12H), 1.56–1.50 (m, 3H), 1.35 (d, J = 11.5 Hz, 6H). ESI-MS: *m*/*z* calculated for C_36_H_46_N_3_^+^: 520.37; found: 520.4.

## 3. Results and Discussion

**Scheme 1:**
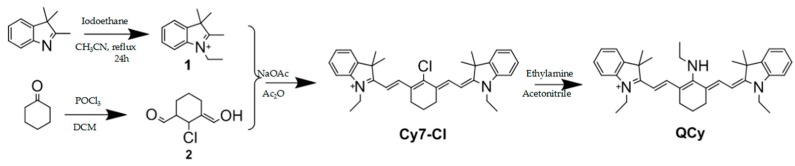
The synthesis of the QCy probe.

The synthesis of the QCy probe is presented in the Experimental Section, and its characterization by ^1^H NMR and MS is presented in the Supporting Information. First, we synthesized 1-ethyl-2,3,3-trimethyl-3*H*-indol-1-ium iodide and 2-chloro-3-(hydroxymethylene)-1-cyclohexene-carboxaldehyde. Then, the two compounds were heated with anhydrous sodium acetate in acetic anhydride solution at 100 °C for 1 h to obtain compound Cy7-Cl. Finally, QCy was obtained by reacting Cy7-Cl with ethylamine in anhydrous acetonitrile (Scheme 1).

### 3.1. Fluorescence and UV-Vis Study of the QCy Probe

**Figure 1:**
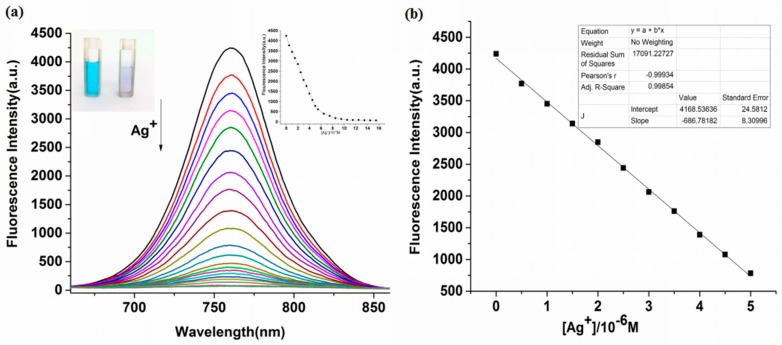
Fluorescence titration studies of QCy. (**a**) Fluorescence spectra of QCy (2.5 µM) upon addition of Ag^+^ (0.0–7.0 µM) in ethanol/phosphate-buffered saline (EtOH/PBS) (1:9 *v*/*v*, pH = 7.0, 10 mM); (**b**) The linear relationship between the fluorescence intensity and the concentration of Ag^+^. The excitation wavelength was 619 nm, and the emission wavelength was 760 nm.

To evaluate the spectroscopic properties of QCy with Ag^+^, a fluorescence titration experiment was conducted in EtOH and PBS (1:9, 2.5 µM, Ph = 7.0). The best ratio of EtOH and the PBS buffer was set at 1:9, to fulfill their cooperative effect (Figure S9). As shown in Figure 1, under 619 nm excitation, QCy in PBS solution showed strong fluorescence emission at 760 nm in the absence of Ag^+^. Under this physiological condition, Qcy exhibits fluorescence quantum yields Φ = 0.42 [26]. With the stepwise addition of Ag^+^ to the QCy solution, the fluorescence intensity at 760 nm consistently decreased. When the concentration of Ag^+^ reached 7.0 µM, the fluorescence of the probe was nearly completely quenched. These fluorescence changes could clearly be observed by the naked eye from blue to red (Figure 1a). Figure 1b shows a good linear relationship between the fluorescence intensity and the concentration of Ag^+^ (R^2^ = 0.9985). Based on the results above, a new complex of QCy–Ag^+^ might have formed, providing the possibility for an Ag^+^ assay.

**Figure 2:**
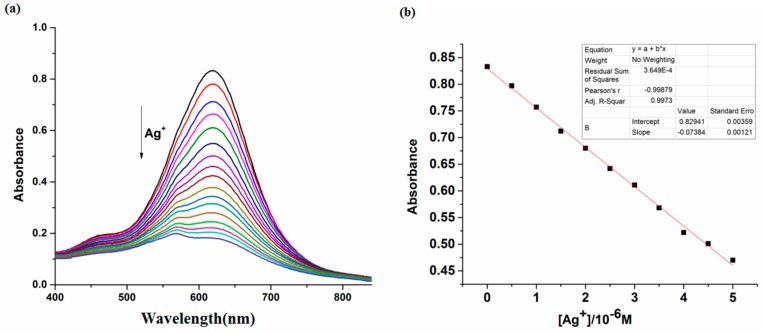
(**a**) The absorbance spectra of QCy (2.5 µM) at different concentrations of Ag^+^ (0.0–7.0 µM) in EtOH/PBS (1:9 *v*/*v*, pH = 7.0, 10 mM). (**b**) The linear relationship between the absorbance intensity and the concentration of Ag^+^.

To further demonstrate the formation of the complex, the UV-vis spectra of QCy upon gradual addition of Ag^+^ were recorded (Figure 2). The absorption spectrum of the probe shows an absorption band at 619 nm (ε = 2.7 × 10^5^ M^−1^ cm^−1^). This band obviously decreased and exhibited a slight blue shift after addition of 7.0 µM Ag^+^; a new peak at 570 nm was also observed (ε = 5.8 × 10^4^ M^−1^ cm^−1^). These results can be explained by an effective energy transfer mechanism. The absorption spectrum of the probe at 619 nm weakens gradually with increasing Ag^+^, which may be attributed to the molecular structure of the QCy probe. The spectroscopic features observed thus far support the hypothesis that an interaction occurs between the QCy probe and Ag^+^. QCy not only works well in a EtOH/PBS (1:9) mixture, but it also exhibits a low detection limit, and near-infrared emission. These properties indicate that QCy can be used as an effective tool for sensing Ag^+^.

### 3.2. Selectivity for Ag^+^ Detection

**Figure 3:**
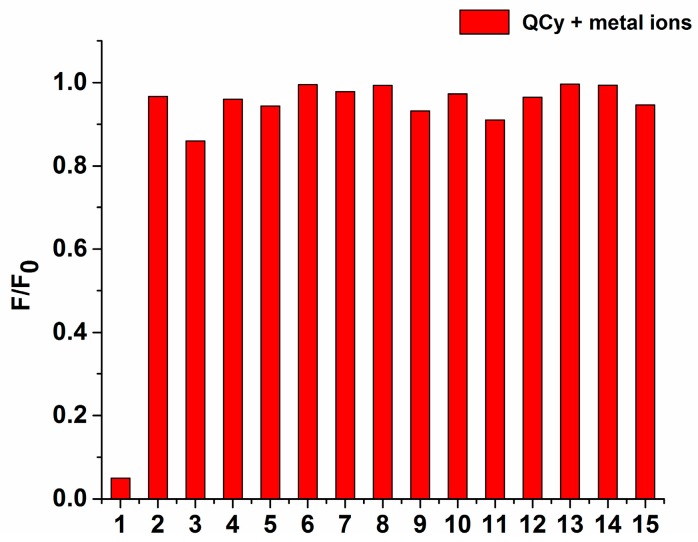
The chart showed an interference test of QCy in the presence of 7.0 µM of different metal ions (Ag^+^, Fe^2+^, K^+^, Na^+^, Ca^2+^, Zn^2+^, Mg^2+^, Pb^2+^, Ni^2+^, Mn^2+^, Cd^2+^, Cu^2+^, Hg^2+^). All studies were acquired in a mixed solvent of EtOH/PBS (1/9, *v*/*v*, pH = 7.0, 10 mM).

A highly selective response to the target species compared with that to other potentially competing species is very important for fluorescent probes, and essential to their potential application in complex biological and environmental samples. The selectivity of QCy toward various metal ions was investigated, and the results are shown in Figure 3. No significant fluorescence change was observed in the presence of common cations, such as Fe^2+^, K^+^, Na^+^, Ca^2+^, Zn^2+^, Mg^2+^, Pb^2+^, Ni^2+^, Mn^2+^, Cd^2+^, Cu^2+^, and Hg^2+^. After the addition of Ag^+^, however, the fluorescence of QCy markedly decreased. The presence of Ag^+^ in the test system produced a significant fluorescence change signal. When the same concentration of other metal ions was added to the sensing system, the change in the fluorescence was negligible. This result clearly indicates that the fluorescent QCy probe has a high affinity to and selectivity for Ag^+^, relative to most other competitive metal ions.

### 3.3. Time Dependence of Ag^+^ Detection

**Figure 4:**
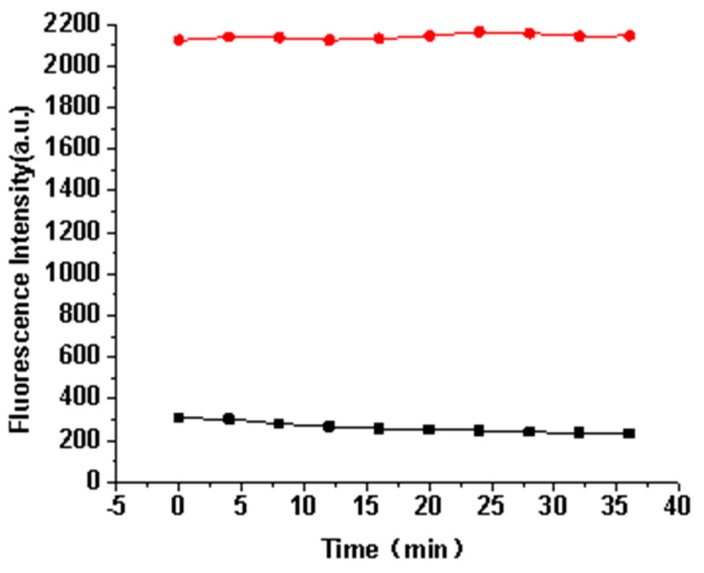
Time-dependent normalized fluorescence changes of QCy (1.0 µM) with Ag^+^ (5.0 µM) (**black**) and without (**red**) at 760 nm in EtOH/PBS (1:9 *v*/*v*, pH = 7.0, 10 mM).

The response time is an important factor for evaluating a fluorescent probe; hence, we further examined the time course of the fluorescence intensity of QCy in the presence of 5 equivalents of Ag^+^ in solution. In EtOH/PBS (1:9 *v*/*v*, pH = 7.0, 10 mM), the fluorescence of QCy at 760 nm exhibited no change in the absence of Ag^+^ over the course of the experimental period. However, its fluorescence was quenched immediately after the addition of 5 equivalents of Ag^+^ (Figure 4). A stable reading could be obtained within less than 1 min, which indicates that the response of the QCy probe toward Ag^+^ is fast. In addition, upon the addition of 5 equivalents of Ag^+^, the fluorescence intensity of the probe not only sharply decreased and plateaued, but it also remained stable for some time. This finding reveals that the probe has good photostability under light irradiation.

### 3.4. The Detection Limit

The detection limit was calculated based on fluorescence titration according to the literature [27]. The detection limit of the probe for Ag^+^ was studied using various concentrations of Ag^+^ (0.0–7.0 µM). The fluorescence intensity at 760 nm was plotted as a function of Ag^+^ concentration in EtOH/PBS (1/9, *v*/*v*, pH = 7.0, 10 mM). We also measured the fluorescence emission spectra of QCy 11 times at each Ag^+^ concentration, to obtain the standard deviation of the blank measurement. To obtain the slope, the fluorescence intensity at 760 nm was plotted against the concentration of Ag^+^. Thereafter, the detection limit was calculated, using the following equation:

Detection limit = 3 σ/k. Where σ is the standard deviation of the solution in the absence of Ag^+^ and k is the slope of equation obtained from plotting the fluorescence intensity versus the concentration of Ag^+^. The detection limit was measured to be 0.03 µM. As such, the QCy probe is highly sensitive toward Ag^+^.

### 3.5. pH Titration and Spectral Responses of the QCy Probe

**Figure 5:**
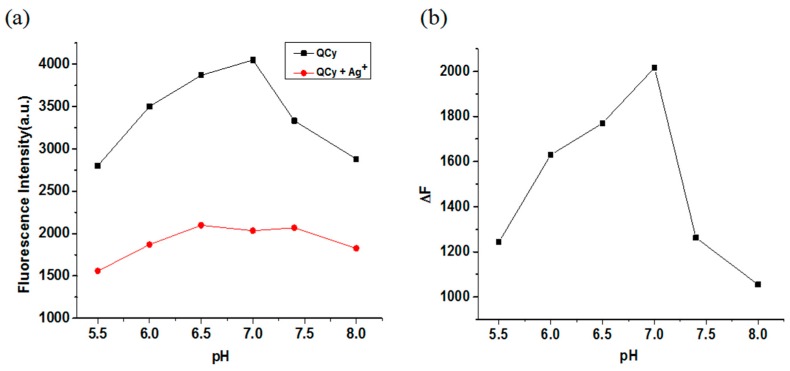
Dependence of Probe QCy with or without Ag^+^ complex fluorescence intensity on pH level.

The pH may present important interferences to the probe; thus, we examined the fluorescence properties of QCy under different solution pH in the absence or presence of Ag^+^. Figure 5a reveals that the fluorescence quenching phenomenon of QCy was obvious in the detection of Ag^+^ at pH 5–8. First, QCy showed strong fluorescence in the absence of Ag^+^ at pH 7.0. In addition, the fluorescence intensity of QCy at other pH values was smaller than that of QCy at pH 7.0. The difference in the fluorescence intensity of QCy in the presence and absence of Ag^+^ was the largest at pH 7.0, which indicates that the fluorescence quenching effect is optimal at pH 7.0 (Figure 5b). The fluorescence intensity initially decreased rapidly (pH = 5.5–7.0), which can probably be attributed to the deprotonated nitrogen in –NHCH_2_CH_3_, and then it drastically decreased (pH = 7.0–8.0), which can be due to the formation of AgOH. These results reveal that QCy may be effective in detecting Ag^+^ under physiological conditions (pH 7.0).

### 3.6. Binding Mechanism

**Figure 6:**
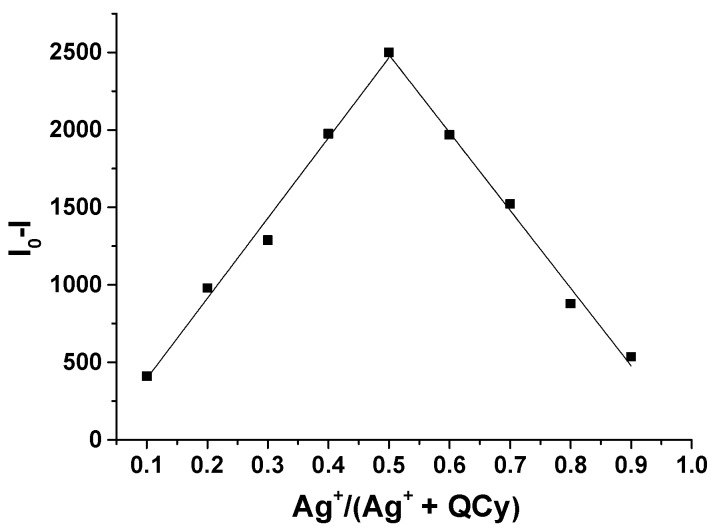
Fluorescence at 760 nm of QCy and Ag^+^ in EtOH/PBS (1/9 *v*/*v*, pH = 7.0, 10 mM), with a total concentration of [QCy] + [Ag^+^] = 10 µM, indicating a 1:1 metal–ligand ratio of the complexation between QCy and Ag^+^.

**Figure 7:**
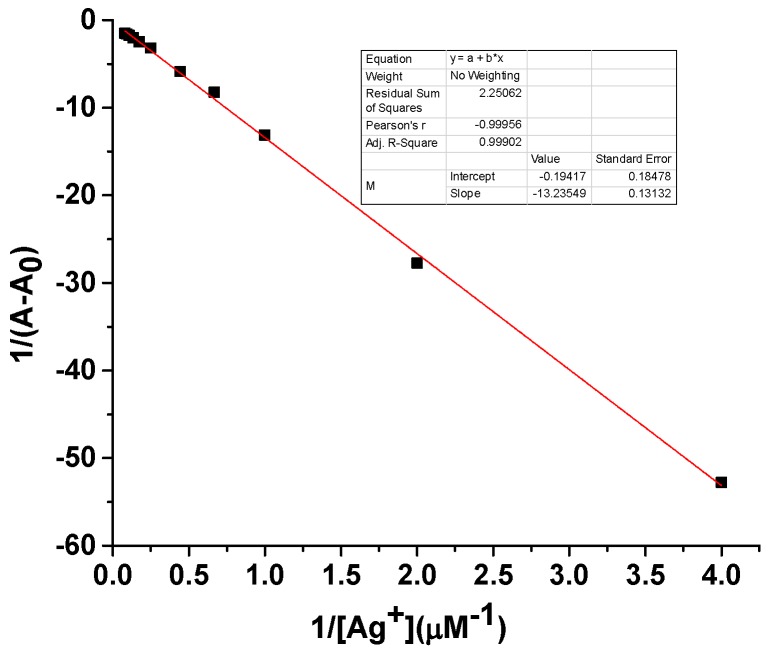
Benesi–Hildebrand plot of QCy with Ag^+^.

In Figure 6, the [QCy/Ag^+^] complex approached its fluorescence quenching maximum at a molar fraction of 0.5, which confirms the 1:1 complexation stoichiometry of the [QCy/Ag^+^] complex, as predicted by Job’s plot analysis [28]. Moreover, according to the Benesi–Hildebrand equation, 1/(A − A_0_) of QCy at 619 nm showed a good linear relationship with 1/[Ag^+^] (R^2^ = 0.9990), and the association constant was calculated to be 1.47 × 10^4^ M^−1^ from the slope and intercept of the equation obtained (Figure 7) [29], This result indicates that the QCy probe and Ag^+^ generate a stable complex in a solution of EtOH and PBS (1:9, pH = 7.0).

**Figure 8:**
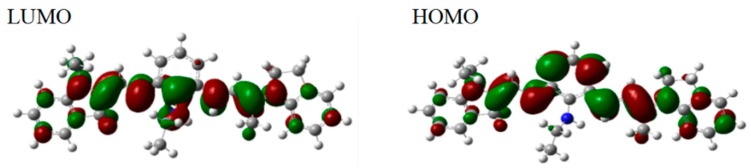
Calculated highest occupied molecular orbital (HOMO) and lowest unoccupied molecular orbital (LUMO) of QCy.

**Figure 9:**
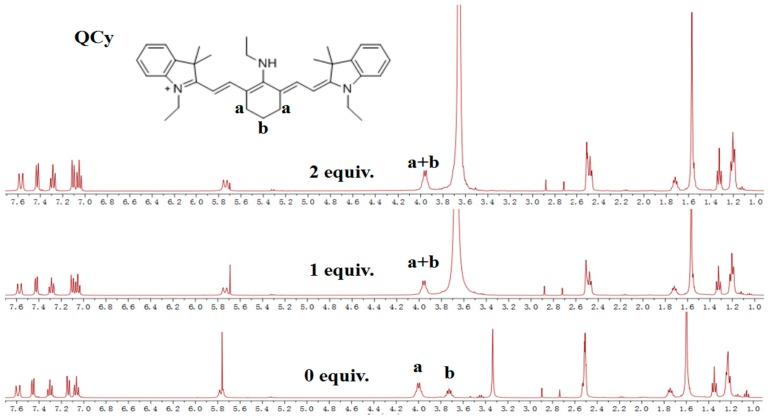
^1^H NMR spectra of QCy obtained upon an incremental addition of Ag^+^ in DMSO-d_6_.

**Figure 10:**
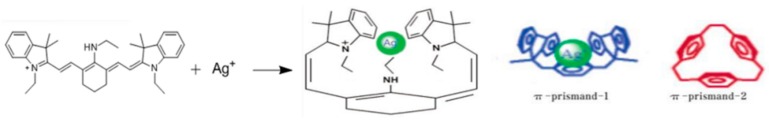
Proposed binding modes of QCy and Ag^+^, and a similar structure of the π-prismand.

To investigate the interaction mode between QCy and Ag^+^, we performed density functional theory calculations. Figure 8 presents the lowest unoccupied molecular orbital (LUMO) and highest occupied molecular orbital (HOMO) of QCy. The LUMO orbital is mainly distributed on the π* conjugated system of the fluorescent matrix [30]. Additionally, the HOMO orbital is completely distributed on its π-conjugated system; thus, the transition corresponds to a π→π* vertical shift of the fluorescent matrix. Ag^+^ induces the fluorescence quenching effect of QCy through intramolecular d–π interactions between the fluorophore and the ions. Also, the amine group of QCy has an electron-donating effect, it can increase the electron cloud density of the conjugated system, and the π-electron cloud of the conjugated system enters the 4d orbital of the silver, thereby making the combination of silver and probe more stable. We can clearly see the significant chemical shifts in the ^1^H NMR signals arising from the a moiety and b moiety, and the slight shift in the chemical shifts of the two indole rings after the addition of Ag^+^(Figure 9). It can been seen that the hydrogen on the molecule has a slight shift to the upfield, which indicates that the molecule forms a strong complex with the silver ion. The movement of hydrogen can be explained by the fact that the electrons of silver ions are shared with the molecules, causing the electron cloud density of the molecules to increase, the shielding effect becomes stronger, so the hydrogen protons are moved to the upfield. The intramolecular d–π interaction of the complex Ag^+^–QCy is very strong, and it results in a decrease in absorption and color change in the solution. Changes in the absorption spectrum, with no new band appearing in the fluorescence spectrum in the present case, suggest that the quenching mechanism is due to the non-fluorescent complex formation between the dye and Ag^+^ in the ground state. In fact, some researchers have confirmed the presence of intramolecular d–π interactions in some coordinated systems. QCy can efficiently bind a single Ag ion due to its ability to readily adopt a π prismand-like conformation through simple C–C bond rotation [31]. The cavity formed by the three walls (two indole rings and one based-cyclohexane ring) of QCy is remarkably similar to that found in the π-prismand, a well-known and effective Ag^+^ acceptor (Figure 10). These observations, along with the UV spectra, support the supposition that an additional d–π interaction exists between QCy and Ag^+^ [32].

### 3.7. Analysis of Ag^+^ in Water Samples

**Figure 11:**
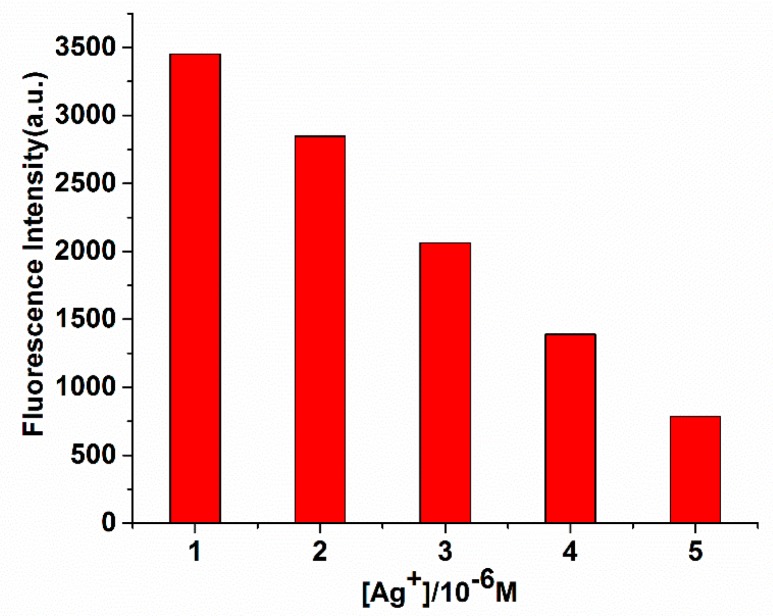
Fluorescence signaling of Ag^+^ by QCy in simulated wastewater. [QCy] = 2.5 µM, [Ag^+^] = 0.0–5.0 µM. All studies were acquired in a mixed solvent of EtOH/PBS (1/9 *v*/*v*, pH = 7.0, 10 mM).

To demonstrate whether the developed probe was applicable to water samples, we prepared simulated wastewater sample solutions containing varying amounts of Ag^+^, and we measured the fluorescence intensities of QCy in these solutions at 619 nm. The simulated wastewater was prepared according to literature examples, and its composition is shown in Table 1. The obtained calibration plot demonstrated a linear relationship between the fluorescence intensity and Ag^+^ concentration in the simulated wastewater samples (Figure 11); the fluorescence emissions are recorded in Figure S10. These observations suggest that the probe can be applied to determine Ag^+^ in wastewater samples. We also investigated whether the probe is applicable to natural samples. Three water samples were collected for this analysis: Tap water from the laboratory (Figure S11), Xuanwu Lake (Figure S12) and Qinhuai River (Figure S13). We measured the contents of silver ions in three water samples by atomic absorption spectrometry, and the result support that the water samples do not contain silver ions (Figure S14). The recovery test was performed by adding a known amount of Ag^+^ to the samples, and the results are shown in Table 2. QCy showed a slightly lower recovery rate in river water than in tap water, likely due to the presence of complex oxides in the river water, which could affect the fluorescence response of QCy to Ag^+^. Overall, QCy can maintain relatively stable fluorescence signals in actual water samples, and it has a high recovery rate for detecting Ag^+^. Considering these findings, the probe developed for Ag^+^ detection is obviously reliable, practical, and economical in operation.

## 4. Conclusions

In summary, a new “turn-off” near-infrared fluorescent probe (QCy) was developed for the recognition of Ag^+^. It displays highly sensitive and selective detection of Ag^+^ over other metal cations, with a low detection limit 0.03 µM. Moreover, the probe has been used for the determination of Ag^+^ in real water samples such as tap water, lake water, and river water, further demonstrating its reliability. The development of the QCy sensor expands the library of available fluorescent probe for detecting Ag^+^. We believe that this fluorescent probe QCy may have potential application for the detection of Ag^+^ in environmental areas.

## Figures and Tables

**Table 1:** Composition of simulated wastewater.

Metal Salt	Concentration (M)	Metal Salt	Concentration (M)
NaClO_4_	2.39 × 10^−3^	Fe(ClO_4_)_3_	5.37 × 10^−5^
KClO_4_	2.81 × 10^−4^	Ni(ClO_4_)_2_	1.36 × 10^−5^
Mg(ClO_4_)_2_	2.88 × 10^−4^	Al(NO_3_)_3_	8.64 × 10^−5^
Ca(ClO_4_)_2_	2.50 × 10^−4^	Mn(ClO_4_)_2_	1.82 × 10^−6^
Cu(ClO_4_)_2_	3.15 × 10^−4^	Pb(ClO_4_)_2_	7.24 × 10^−6^
Zn(ClO_4_)_2_	1.07 × 10^−5^		

**Table 2:** Determination results of Ag^+^ in the environmental samples by fluorescent method using QCy (2.5 μM).

Sample	Found (μM)	Added Silver Ions (μM)	Detected Silver Ion Content (μM)	Recovery Rate (%)	Standard Deviation
1	No detected	5	4.97	99.4	0.094
2	No detected	5	4.93	98.6	0.063
3	No detected	5	4.92	98.4	0.076

1 Tap water of laboratory. 2 Xuanwu Lake. 3 Qinhuai River.

## Supplementary Materials

The following are available online at http://www.mdpi.com/1424-8220/19/2/247/s1, Table S1: Comparison of the QCy probe with some other chemodosimeters for Ag^+^. Figure S1: ^1^H NMR spectrum of Compound **1** in d-chloroform; Figure S2: ^13^C NMR spectrum of Compound **1** in d-chloroform; Figure S3: Mass spectrum of Compound **1**; Figure S4: ^1^H NMR spectrum of Cy7-Cl in d-chloroform; Figure S5: ^13^C NMR spectrum of Cy7-Cl in d-chloroform; Figure S6: Mass spectrum of Cy7-Cl; Figure S7: ^1^H NMR spectrum of QCy in d-chloroform; Figure S8: Mass spectrum of QCy; Figure S9: The influence of different proportions between EtOH and PBS. The test conditions: different proportions of EtOH and PBS (from 10:0 to 0:10); Figure S10: Fluorescence emissions of QCy with the addition of Ag^+^ in simulated wastewater. [QCy] = 2.5 µM, [Ag^+^] = 0.0–5.0 µM; Figure S11: Fluorescence emissions of QCy with the addition of Ag^+^ in Tap water of laboratory with three times. [QCy] = 2.5 µM, [Ag^+^] = 5.0 µM; Figure S12: Fluorescence emissions of QCy with the addition of Ag^+^ in Xuanwu Lake with three times. [QCy] = 2.5 µM, [Ag^+^] = 5.0 µM; Figure S13: Fluorescence emissions of QCy with the addition of Ag^+^ in Qinhuai River with three times. [QCy] = 2.5 µM, [Ag^+^] = 5.0 µM; Figure S14: The report of Silver ion content in three water samples by atomic absorption spectrometry.
