# Supplementary material for: A Sensitive Near-Infrared Fluorescent Probe for Detecting Heavy Metal Ag+ in Water Samples
Source: Sensors (Basel). 2019 Jan 10;19(2):247. doi: 10.3390/s19020247 (PMC6358871; doi:10.3390/s19020247)
Supplement: Supplementary file 1 [file sensors-19-00247-s001.pdf]

sensors

Supplementary

A Sensitive Near-Infrared Fluorescent Probe for Detecting Heavy Metal Ag^+ in Water Samples

Yawen Zhang [1], Aiying Ye [1,2], Yuewei Yao [1] and Cheng Yao [1,*]

[1] College of Chemistry and Molecular Engineering, Nanjing Tech University, Nanjing 210000, China; 18761605638@163.com (Y.Z.); 8000000683@czie.edu.cn (A.Y.); yaoyw89@163.com (Y.Y.)
[2] Changzhou Vocational Institute of Engineering, Changzhou 213100, China
* Correspondence: yaocheng@njtech.edu.cn; Tel.: +86-137-0515-8296

Received: 24 December 2018; Accepted: 7 January 2019; Published: date

Table S1. The comparison of this probe with some other chemodosimeters for Ag^+.

Probe	λ_{em} (nm)	Detection Limit (μM)	Interferents
Ref 6	525	5	no
Ref 9	535	7.2	no
Ref 12	470	0.34	Hg^+
Ref 19	525	0.65	no
Ref 18	525	0.09	no
Ref 20	485	0.2	no
Ref 21	490	0.76	no
QCy	760	0.03	no

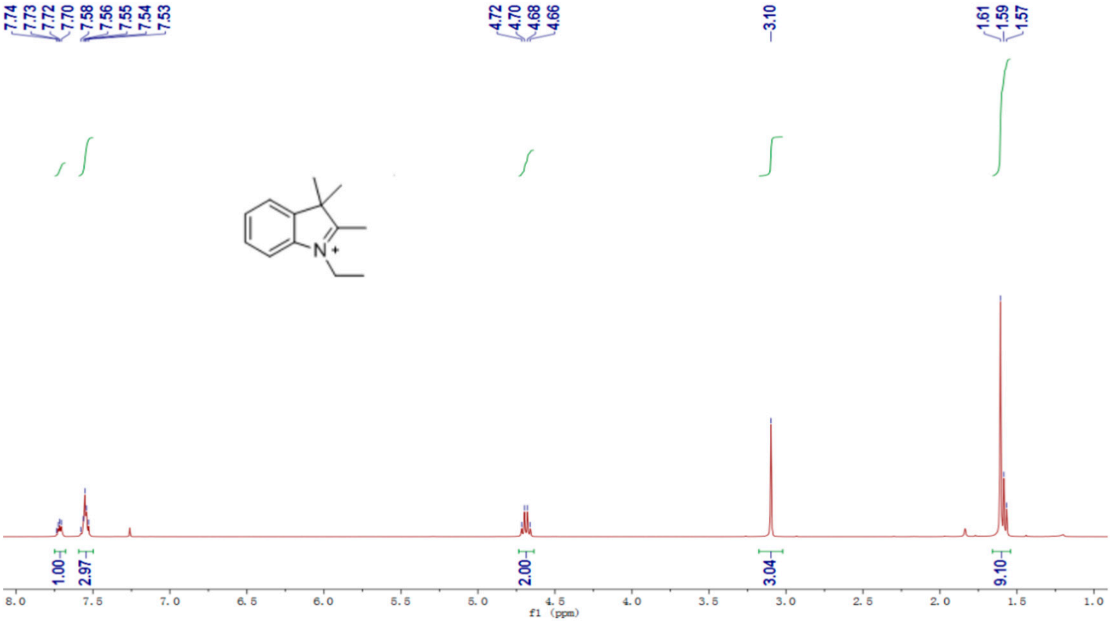

Figure S1. ^1H NMR spectrum of Compound **1** in d-chloroform.

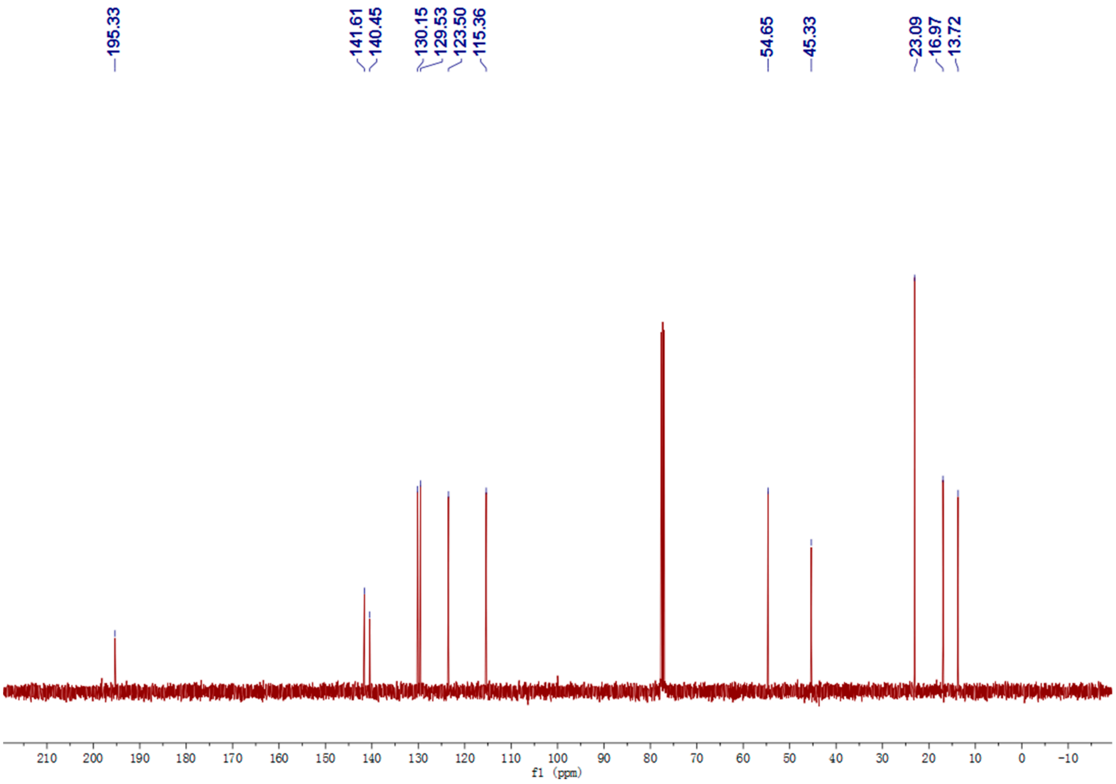

Figure S2. ^{13}C NMR spectrum of Compound **1** in d-chloroform.

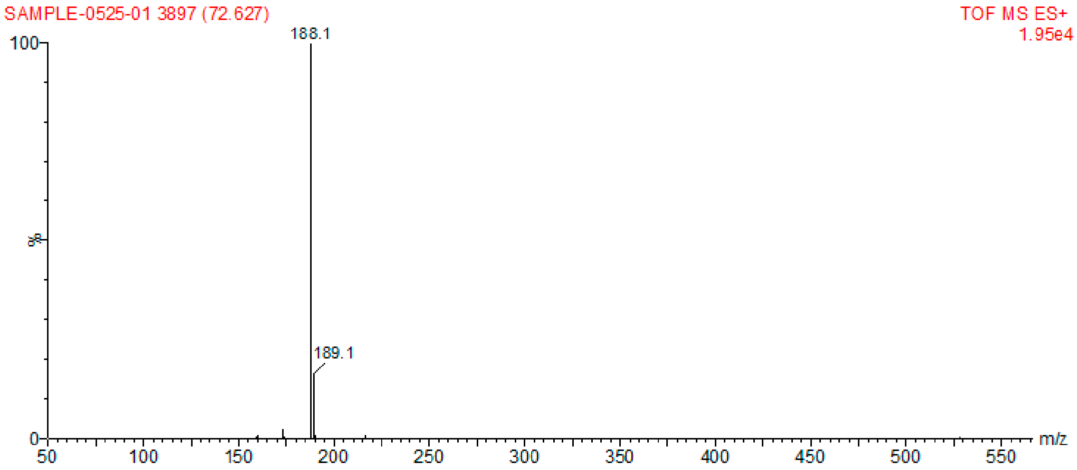

Figure S3. Mass spectrum of Compound **1.**

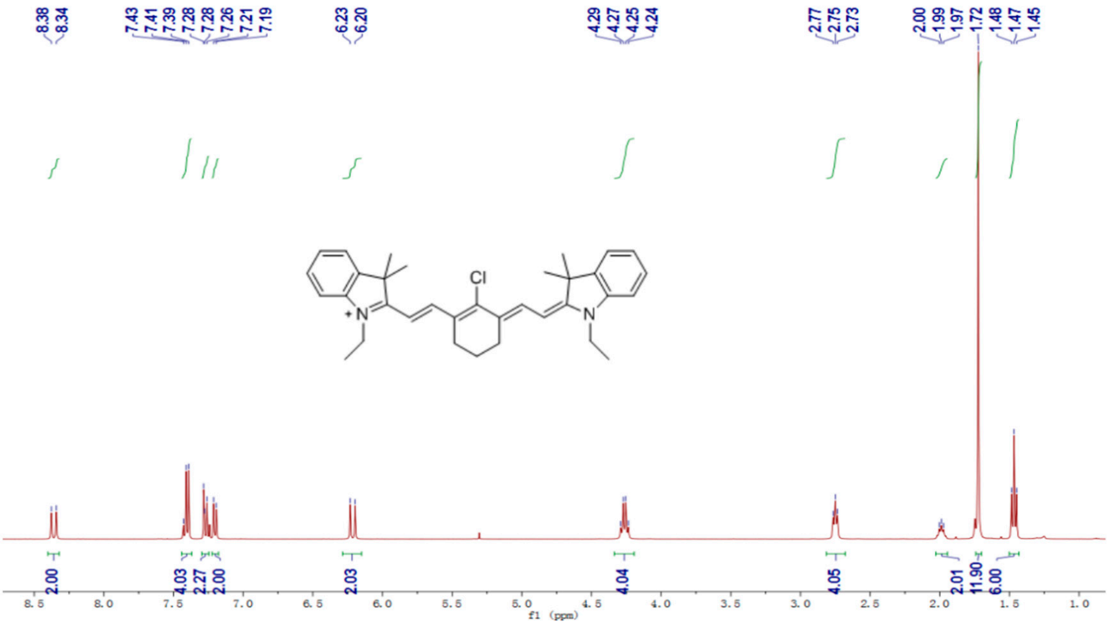

Figure S4. ^{1}H NMR spectrum of Cy7-Cl in d-chloroform.

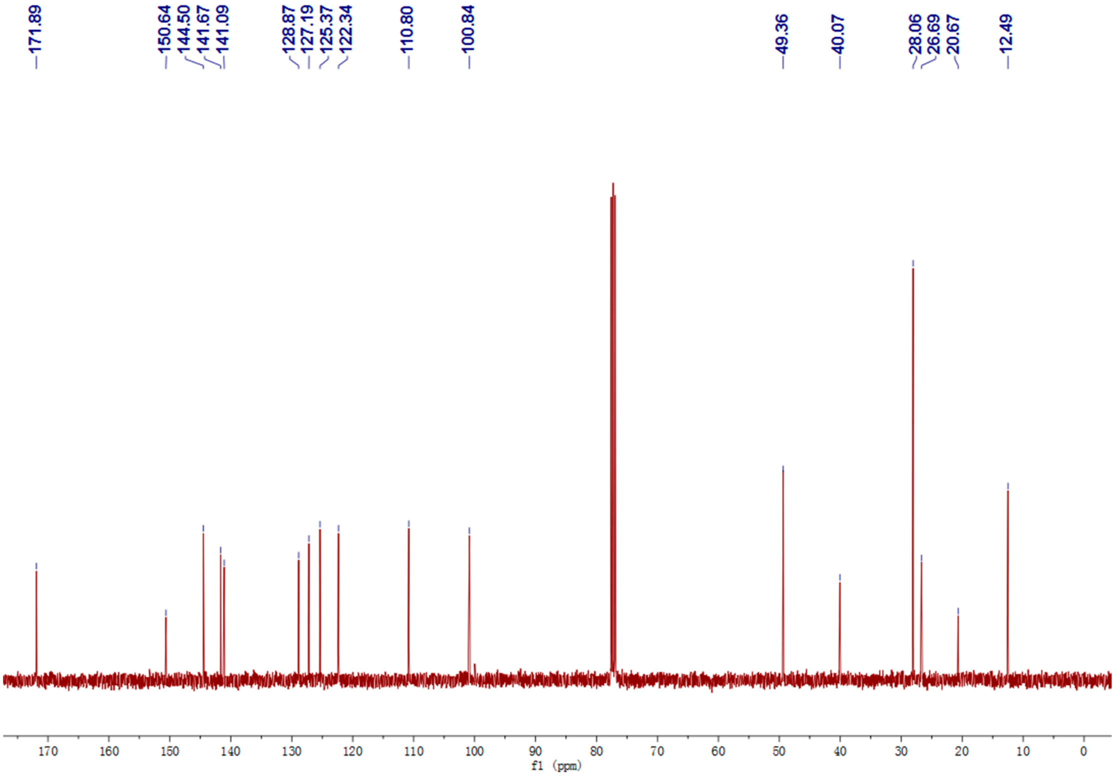

Figure S5. ^{13}C NMR spectrum of Cy7-Cl in d-chloroform.

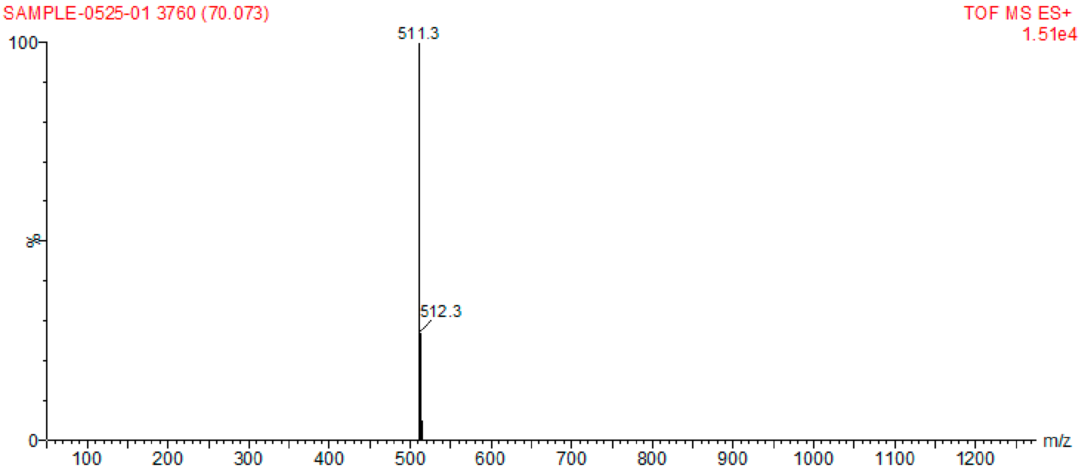

Figure S6. Mass spectrum of Cy7-Cl.

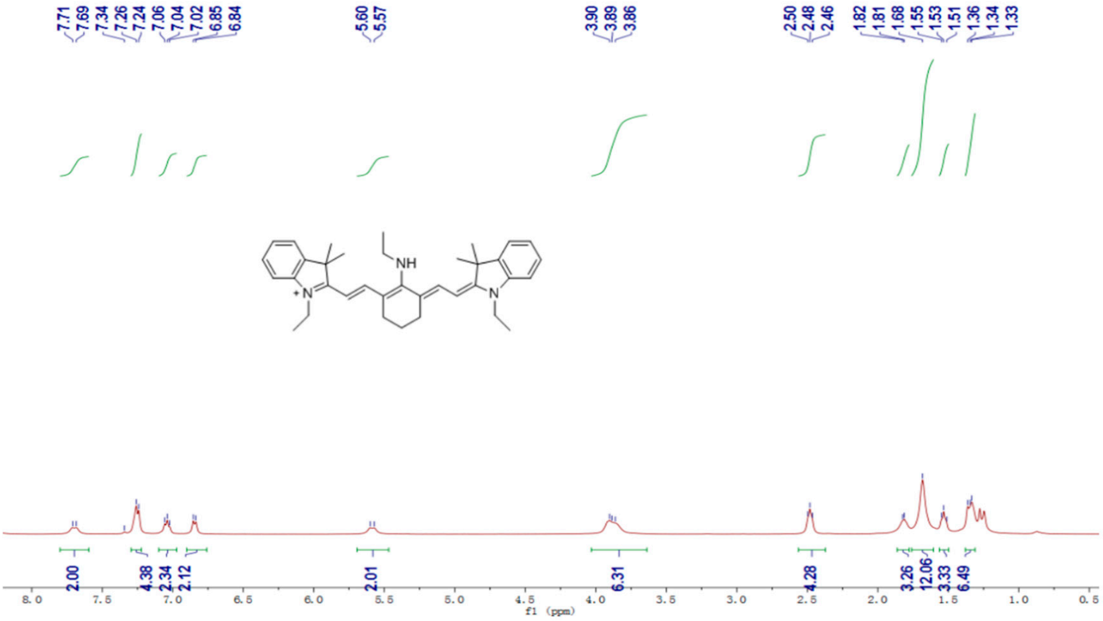

Figure S7. ^{1}H NMR spectrum of QCy in d-chloroform.

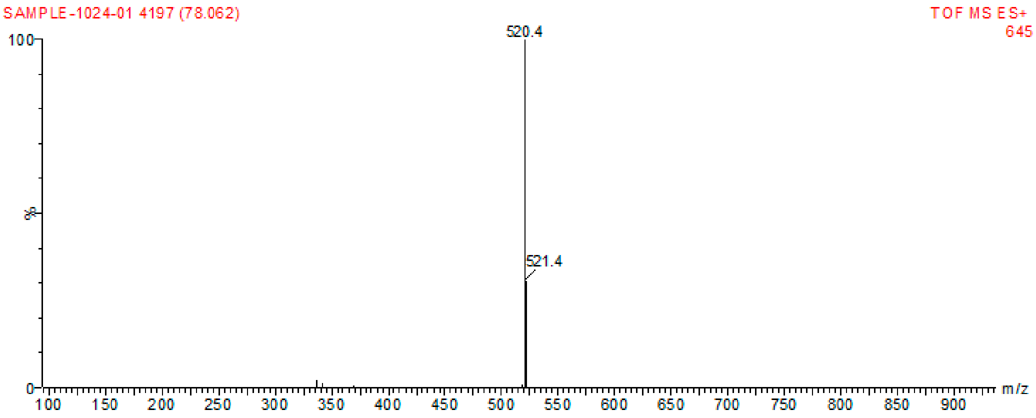

Figure S8. Mass spectrum of QCy.

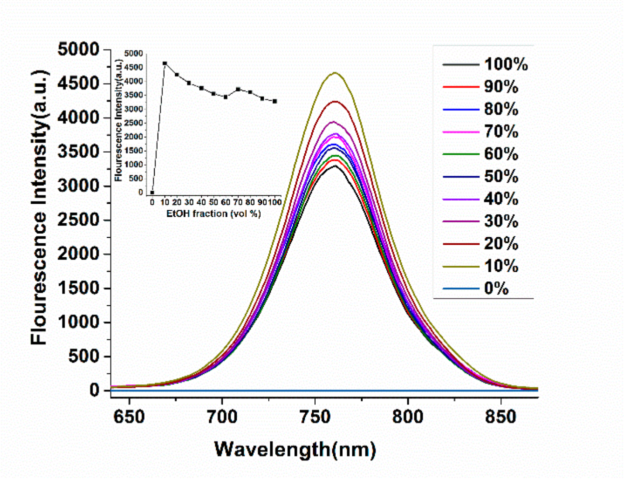

Figure S9. The influence of different proportions between EtOH and PBS. The test conditions: different proportions of EtOH and PBS (from 10:0 to 0:10).

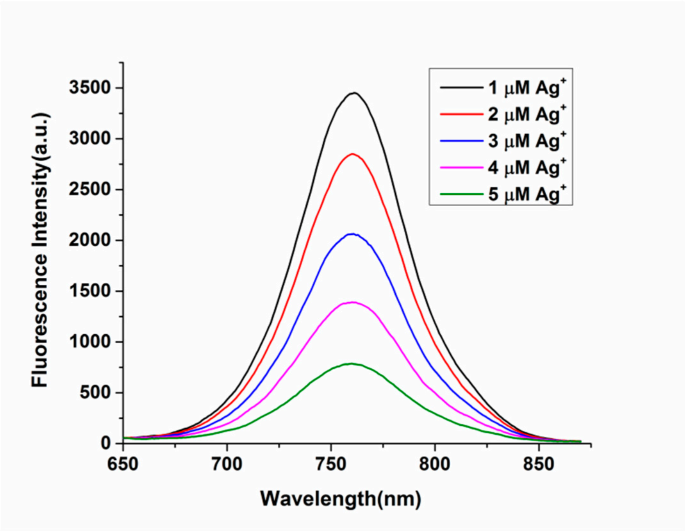

Figure S10. Fluorescence emissions of QCy with the addition of Ag^+ in simulated wastewater. [QCy] = 2.5 μM, [Ag^+] = 0.0–5.0 μM.

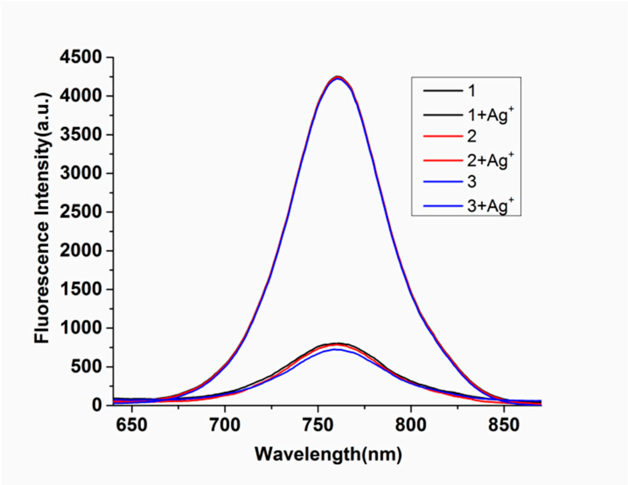

Figure S11. Fluorescence emissions of QCy with the addition of Ag^+ in Tap water of laboratory with three times. [QCy] = 2.5 μM, [Ag^+] = 5.0 μM.

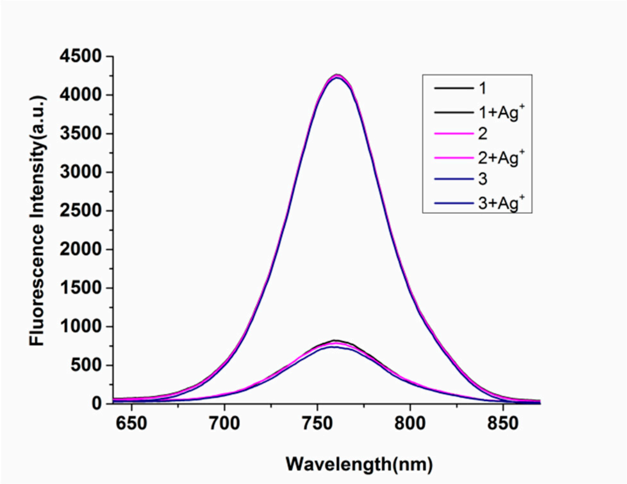

Figure S12. Fluorescence emissions of QCy with the addition of Ag^+ in Xuanwu Lake with three times. [QCy] = 2.5 μM, [Ag^+] = 5.0 μM.

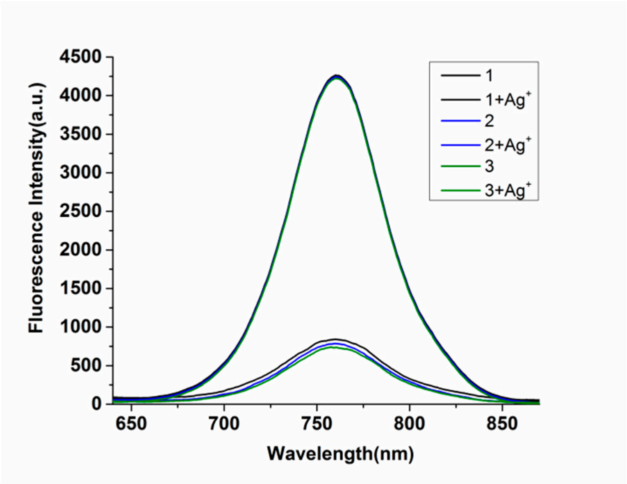

Figure S13. Fluorescence emissions of QCy with the addition of Ag^+ in Qinhuai River with three times. [QCy] = 2.5 μM, [Ag^+] = 5.0 μM.

20190104-Ag.wvq. 所有数据报告，

Ag 328.068 校正 (mg/kg) 2019/1/4, 13:56:41 相关系数: 0.995579

标签	标记	Int.(c/s)	标样浓度。	计算浓度。	错误	%误差
标准 1		492.186	0.000000	-0.251383	-	-
标准 2		61188.7	2.00000	2.62184	0.621842	31.1
标准 3		85448.7	4.00000	3.77025	-0.229747	-5.7
标准 4		128911	6.00000	5.82765	-0.172355	-2.9
标准 5		170650	8.00000	7.80350	-0.196501	-2.5
标准 6		221871	10.0000	10.2281	0.228144	2.3

曲线类型: 线性 等式: y = 21124.9 x + 5802.6

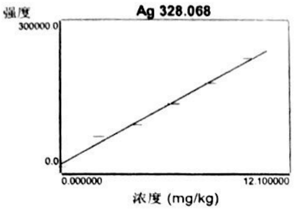

1 (样品) 2019/1/4, 13:59:20 试管 7

重量: 1 体积: 1 稀释: 1

标签	各次浓度
Ag 328.068	-0.228976u

标签	溶液浓度。	单位	SD	%RSD	Int.(c/s)	计算浓度。	DF
Ag 328.068	-0.228976uv	mg/kg	0.000000	0.0	965.544	-0.228976 mg/kg	1.00000

2 (样品) 2019/1/4, 14:00:05 试管 8

重量: 1 体积: 1 稀释: 1

标签	各次浓度
Ag 328.068	-0.238712u

标签	溶液浓度。	单位	SD	%RSD	Int.(c/s)	计算浓度。	DF
Ag 328.068	-0.238712uv	mg/kg	0.000000	0.0	759.863	-0.238712 mg/kg	1.00000

3 (样品) 2019/1/4, 14:00:50 试管 9

重量: 1 体积: 1 稀释: 1

标签	各次浓度
Ag 328.068	-0.239394u

标签	溶液浓度。	单位	SD	%RSD	Int.(c/s)	计算浓度。	DF
Ag 328.068	-0.239394uv	mg/kg	0.000000	0.0	745.445	-0.239394 mg/kg	1.00000

Figure S14. The report of Silver ion content in three water samples by atomic absorption spectrometry.
